# Role of Brain Elastography in the Neonatal Setting: State of the Art of Ultrasonographic Techniques and Future Perspectives

**DOI:** 10.3390/children11070752

**Published:** 2024-06-21

**Authors:** Fiammetta Piersigilli, Francesca Campi, Immacolata Savarese, Giulia Iacona, Cinzia Auriti, Andrea Dotta, Annabella Braguglia, Matteo Garcovich, Iliana Bersani

**Affiliations:** 1 Section of Neonatology, Cliniques Universitaires Saint Luc, Université Catholique de Louvain, 1200 Bruxelles, Belgium; 2 Department of Medical and Surgical Neonatology, Bambino Gesù Children’s Hospital, 00165 Rome, Italyiliana.bersani@opbg.net (I.B.); 3 Department of Medical and Surgery, Imperial College London, London SW7 2AZ, UK; 4 CEMAD Digestive Disease Center, Fondazione Policlinico Universitario “A. Gemelli” IRCCS, Università Cattolica del Sacro Cuore, 00136 Rome, Italy

**Keywords:** neonate, brain, elastography, ultrasound, elasticity, stiffness

## Abstract

Magnetic resonance imaging is currently used in the neonatal setting for assessing features of the neonatal brain. However, its utilization is constrained by logistic, technical, or clinical challenges. Brain elastography is a new research technique which enhances the diagnostic capability of traditional imaging, and can be paired with both ultrasonography and magnetic resonance imaging. In particular, brain elastography adds objective and quantitative information to traditional imaging by detecting differences in tissue elasticity/stiffness, which may represent a surrogate marker of the physiologic and pathologic features of the neonatal brain. To date, very limited experience exists about the use of brain elastography specifically in the neonatal setting. The aim of the present review was to describe the most recent information about the feasibility and diagnostic accuracy of brain ultrasound elastography (USE) in neonates, and to provide information about the possible future applications and perspectives of brain elastography.

## 1. Introduction

The evaluation of cerebral injury in the perinatal period relies on cerebral ultrasonography and magnetic resonance imaging (MRI), whilst computed tomography is generally avoided due to radiation exposure. Cerebral ultrasonography is frequently used in the neonatal setting to assess for brain injury and allows for rapid and low-cost bedside imaging without radiation. Its limitations include the need for adequate operator experience, lack of exact quantification, limited functional information, low ability in the detection of subtle lesions and low prognostic value compared with MRI. MRI is considered the gold standard imaging method, but its use in neonates may be limited due to both logistic and clinical issues, such as difficult accessibility, costs and need to sedate and transport unstable patients to the radiology ward [1,2].

New techniques such as elastography have been developed with the aim of enhancing the diagnostic capability of traditional imaging. Elastography can be paired with both ultrasound and MRI imaging, and in children, is used to investigate multiple organs including the gastrointestinal system (mostly the liver), musculo-skeletal system and central nervous system [1,3,4,5]. Regarding the latter, clinical experience with brain elastography is still at the very beginning [1,4]. Brain elastography’s main advantage is the ability to add objective and quantitative information to traditional neuroimaging by detecting differences in brain mechanical properties. In the last few years, various investigators addressed the feasibility of brain elastography to explore and map brain stiffness both in animals [6,7,8] and in humans [1,4,9,10,11,12,13,14,15,16,17,18,19,20,21]. The use of brain elastography in the neonatal setting is at a very initial phase and only approved in the research setting, but despite the limited experience, such technique seems to deserve special attention. In particular, during the last few years, some authors provided evidence about the utility of brain ultrasound elastography (USE) as a diagnostic tool in neonates, whilst, to the best of our knowledge, no studies addressed the role of magnetic resonance elastography (MRE) in the neonatal period. The aim of the present state-of-the art review was to describe the most recent information about the feasibility and diagnostic accuracy of brain USE, specifically in the neonatal life period, and to provide information about brain elastography’s possible future applications and perspectives.

## 2. Ultrasound Elastography

Many authors defined elastography as an acoustic palpation technique which allows a “virtual touch” of tissues. USE provides non-invasive assessment of the mechanical characteristics of tissues and investigates their mechanical properties by providing a measure (i.e., “modulus”) of how easy it is to deform a material when a stress is applied [22,23,24]. More specifically, easily deformable materials have a low modulus, whilst harder materials have a high modulus. All elastography systems currently available on the market measure some aspect of tissue displacement, but they differ in what and how this is measured. Briefly, elastographic techniques provide information about tissue deformation in one out of three different ways: it may be imaged directly (i), converted to strain (ii), or used to detect the time of the arrival of shear waves, and hence their speed (iii) [22,23,24].

(i) Tissue displacement estimation without further processing: Acoustic Radiation Force Impulse (ARFI) imaging is a qualitative method assessing tissue elasticity by means of conventional ultrasound devices and allowing for the B-mode guidance of tissue stiffness measurement. ARFI imaging is achieved by exposing the target tissue to acoustic radiation force and assessing tissue displacement along the axis of the ultrasound beam. Although displacement itself is a quantitative measurement (units of μm), image brightness is typically scaled between soft (bright) and hard (dark). It is noteworthy that stiffness is not only governed by the tissue’s mechanical properties, but that it is also governed by geometrical factors such as the area of the cross-section of the tissue sample over which the force is applied.
(ii) Tissue strain: “Strain elastography” is a qualitative quasi-static method. Imaging is generated by applying a force by either active external displacement of the tissue surface or passive internal physiologically induced tissue motion (e.g., cardiovascular motion). Softer tissues are more compressible and characterized by higher strain, whilst harder tissues are less compressible and characterized by lower strain.
(iii) Dynamic propagation of shear waves: The display of shear wave speed is calculated by using the time varying displacement data to measure the arrival time of a shear wave at various locations. These methods are generally classified as shear wave elastography (SWE), and include transient elastography (TE), point shear wave elastography (p-SWE) and multidimensional SWE. The shear deformation generated locally by a dynamic force and momentarily within tissue will propagate as a shear wave and will reach a distant location after a time determined by the shear wave speed, which will be displayed in units of meters per second (m/s). Alternatively, assuming that shear deformation does not vary with the magnitude or frequency of the applied force, or with position and direction in the tissue, shear wave speed may be converted to either Young’s modulus E or shear modulus G, which are expressed in units of kilopascal (kPa) [25].

TE is performed without B-mode control and is based on low-frequency vibration pushes. The velocity of the wave is directly related to tissue stiffness. The main limit of TE is that operators are not able to select the optimal areas to investigate, i.e., that unreliable results might be achieved if unwanted components are included in the analysis. 

p-SWE is based on shear wave speed measurement at a location using ARFI. The operator can select the depth at which tissue elasticity is evaluated by placing a fixed “region of interest” (ROI) in the chosen area and usually performing 5–10 measurements. This technique has also been addressed in the literature as ARFI or Virtual Touch Quantification (VTQ) because it was the first method commercially available. Lately, most ultrasound vendors have implemented the p-SWE technique in their machines.

Multidimensional SWE is based on the transmission of an ARFI from the transducer into the investigated tissue. SWE can produce two- or three-dimensional quantitative images of shear wave speed (2D-SWE and 3D-SWE). Of the multiple elastographic techniques developed in recent years, 2D-SWE seems the most accurate, reliable, quantitative, and user-friendly technique [1]. After a B-mode scan to select the most appropriate area, a series of push pulses create plane shear waves which, propagating over the scanned zone, are progressively distorted by tissue heterogeneities and enable the creation of a complete mapping of tissue elasticity. With 2D-SWE working continuously and not with single-shot emissions (as for pSWE), the SWE acquisition is continued for 4–5 s to achieve the homogeneous color filling of the SWE ROI [25]. Ultimately, multimodal SWE has been developed by many ultrasound manufacturers, although only a few have achieved (or even started) clinical validation.

## 3. Brain Elastography in the Neonatal Setting

Currently, the Food and Drug Administration approval for ultrasound elastography in children is limited to solid abdominal organs, and the use of brain USE is still experimental and requires Institutional Review Board approval and consent [4]. 

Brain USE has multiple relevant advantages. (i) It is an easily reproducible, portable, bedside technique. (ii) In contrast with routine imaging, it provides objective and quantitative information about brain mechanical properties. (iii) It provides basal values of brain stiffness/elasticity and is able to monitor changes in each specific region throughout time. (iv) It is a non-invasive technique with good safety profile. (v) It generally does not require sedation [1,4,5]. In particular, the use of brain USE might be of help to characterize, in more detail, the physiologic, age-dependent brain characteristics existing between term and preterm neonates. Moreover, being able to detect precocious and subtle modifications in tissue mechanical features, which may alter brain elastic properties, brain elastography may represent a surrogate diagnostic and monitoring marker for pathologic disorders including perfusion changes, cytotoxic/vasogenic edema, hemorrhages, hydrocephalus and intracranial pressure [1,4,5,7,8,9,10,11,12,13,14,15,16,17,18,19,20,21].

Although overall considered a safe diagnostic technique [10,11,12,26,27,28], the brain USE safety profile requires further investigations. In particular, the thermal index used by elastography is higher compared to traditional ultrasonography [29], due to the higher intensity as well as the longer duration of its acoustic pulses. Elastography-induced changes in temperature are within the allowed limits [30], but there are still no guidelines specifically addressing neonatal BE, especially considering that the bone surrounding the anterior fontanel is characterized by greater heat absorbance [31]. In a mice neonatal model, Li et al. investigated the brain histologic effects of SWE following 10, 20 or 30 min exposure [32]. The authors reported no mechanical/thermal damage after 24 h and after 3 months. They described an increased expression of proteins associated with neurite extension and synaptic plasticity 24 h after exposure to SWE, which was still not confirmed after 3 months. These results suggest that brain elastography might only transiently influence neuronal development with no long-term effects [32]. Although the scanning times required for brain elastography in clinical practice would last only a few seconds and would be much shorter than those assessed by the authors, these data underline the importance of minimizing the examination times. Recently, the same group also investigated the effect of SWE irradiation on the brain expression of the brain-derived neurotrophic factor (BDNF) and SWE effects on neuronal apoptosis in a model of neonatal mice (<48 h of life). The authors found that SWE irradiation for more than 10 min temporarily downregulated the expression of BDNF, although such effect disappeared within 24 h after exposure. Moreover, SWE did not significantly affect the number of apoptotic neurons [33]. Taken together, these data support the good safety profile of neonatal brain elastography, with no reported long-term side effects. Nevertheless, considering the differences between mice and human neonates in terms of brain volume and skull hardness, studies investigating whether such biologic effects are confirmed in humans are required, and brain elastography should be used cautiously and with minimal exposure time [33].

To date, limited clinical experience exists about the use of brain USE, specifically in the neonatal period (Table 1). 

Since physiologic cerebral neurodevelopmental processes may influence brain stiffness/elasticity in the neonatal period, brain elastography might help to describe the physiologic age-dependent developmental features of the neonatal brain, both in preterm and term neonates. Brain elastography may also assign specific stiffness values for each brain anatomic location, characterized by peculiar structure and mechanical properties. Brain USE technical feasibility and reproducibility in the neonatal setting is satisfactory and, when assessed, brain elastography evaluations showed good interobserver agreement [11,12,26,27,28] (Table 1). The necessary prerequisites for a valid brain elastography investigation are a sufficient fontanel space for the examination and infants being calm during the examination. Moreover, neonatal brain elastography values may vary according to the different technical equipment used for the investigation, underlying the need for the standardization and proof of concept studies.

Importantly, every diagnostic brain USE needs proper age- and region-specific reference curves to be reliable. Reference ranges have already been described in adults [34,35,36,37], and, in recent years, some researchers have also begun to provide possible brain elastography reference values, also for the neonatal period, by assessing neonates with normal brain parenchyma [10,11,12,26]. Su et al. investigated 41 neonates with no brain injury at different gestational ages by using the ARFI technique (VTQ). The authors established baseline values for this elastographic technique both in term and preterm infants, and found that full-term newborns showed higher elasticity than preterm ones. Such an increase in elasticity may be related to the increased myelination occurring during normal brain development [10]. Kim et al. assessed neonatal brain features by using semi-quantitative strain elastography in 21 neonates with a mean gestational age of 34 weeks (28–40 weeks). The authors assigned a 5-point color scale to different intracranial regions (ventricle, periventricular white matter, caudate, subcortical, cortical gray matter, and subdural space). They found that cortical gray matter had higher elasticity than all the other regions, that the caudate had lower elasticity compared to periventricular white matter and cortical gray matter, that the periventricular white matter showed higher elasticity compared to subcortical white matter (although, with similar median values), and that the caudate and subcortical white matter showed comparable values. No sex-related differences in brain elasticity were found [11]. Albayrak et al. investigated the mechanical properties of neonatal brain by using 2D-SWE in 83 neonates (44 term and 39 preterm neonates), with the aim to investigate possible stiffness differences between groups. Neonates with any cerebral hemorrhage and hydrocephalia were excluded. The authors reported that brain parenchymal stiffness values measured from the thalamus and periventricular white matter were significantly lower in preterm than in term neonates, and that the periventricular white matter stiffness was lower than the thalamus one, both in preterm and term newborns [12]. Garcés-Iñigo et al. prospectively included 55 healthy term neonates between the second and third postnatal days and investigated the thalamus and corona radiata to measure stiffness by another 2D-SWE. The authors found that thalamus elasticity was lower compared to corona radiata (1.17 m/s vs. 1.60 m/s, mean velocity values) in healthy term neonates and that the sex and type of delivery did not influence the results [26].

Deviations from known reference values of neonatal brain USE may also provide information about brain abnormalities and predict prognosis. To date, only a few authors investigated the role of neonatal brain elastography as a diagnostic tool in case of underlying pathologic conditions both in animal models [38,39] and humans [27,28]. 

In neonatal animal models, some authors recently investigated the diagnostic ability of brain elastography in the assessment of brain injury following hypoxic–ischemic damage. Wang et al. investigated the correlation between brain histopathology and ARFI elastographic features in a rat model of hypoxic ischemic brain damage. The authors reported increased stiffness values after exposure to the hypoxic injury and increased results in the asphyxia group compared to controls. Such data showed a consistent correlation with brain histopathological patterns [38]. Similarly, Zhu et al. also explored whether an elastographic assessment by the ARFI technique allowed for a precocious identification of brain damage in a mice model of neonatal hypoxic–ischemic injury. The authors reported differences in shear wave velocity which were in agreement with brain histopathologic modifications according to disease severity, suggesting that such a technique may be of help for an early stratification of neonatal hypoxic–ischemic brain injury [39].

In human neonatal populations, brain elastography’s ability as a diagnostic tool for pathologic conditions remains largely underexplored thus far. El-Ali et al. assessed whether the 2D-SWE of brain parenchyma was able to detect intracranial pathology in newborns. The study included 70 neonates with normal examinations and 8 with intracranial pathology for analysis. The authors found that neonates with large intraparenchymal hemorrhages had a tendency towards an increased white matter and deep gray nuclei stiffness compared to full-term infants with a normal ultrasound [28]. In contrast with the data by Albayrak et al., the authors did not highlight differences according to gestational age, but since the examination was performed at about 5-6 weeks of life, any difference in brain stiffness related to gestational age might have been masked by the ongoing myelination process [28]. 

Dirrichs et al. prospectively analyzed the feasibility of 2D-SWE in the assessment of brain mechanical features in 184 infants, of whom 56 had hydrocephalus and 110 were healthy, with a mean age of 12 weeks (ranging from 1 day to 12 months). The authors found that SWE was feasible in the assessment of infants with increased intracranial pressure and hydrocephalus. Their results also showed that SWE values and, thus, brain parenchymal rigidity, were significantly higher in patients with hydrocephalus compared to healthy infants and that a direct correlation existed between stiffness values and increased cerebral pressure values in a subgroup of patients with hydrocephalus. This suggests that SWE may represent a surrogate marker for increased intracranial pressure in infants [27]. In summary, what emerged from the abovementioned preliminary studies is that the elastographic features of the neonatal brain vary according to different brain regions [11,12,26] and the different age/developmental phase, and that the presence of pathologic conditions seems to affect cerebral mechanical properties. 

As for neonatal brain elastography, the role of brain elastography in prenatal investigations has also been minimally investigated [40,41]. Studies investigating the diagnostic ability of prenatal brain elastography may provide detailed clarifications about physiologic brain features according to the gestational age or abnormal cerebral patterns under pathologic conditions. Possible limitations for prenatal brain elastography might be the low sensitivity in detecting different brain regions due to shorter shear wave propagation and detection depth [4,40].

In conclusion, brain USE is feasible in the neonatal setting and, apparently, characterized by a good safety profile. Despite promising results with this relatively new diagnostic technique, however, the overall number of neonates included in the currently available studies is still low and different ultrasound elastographic techniques have been used in studies (Table 1). Therefore, any conclusive consideration about neonatal brain USE diagnostic accuracy is still premature and larger, well-designed studies are warranted to fill such important gaps in knowledge. In particular, future studies should achieve the following: (i) confirm neonatal reference ranges for each elastographic technique, enabling the creation of a complete mapping of brain tissue elasticity; (ii) clarify the effect of gestational and postnatal age on brain elastography results; (iii) describe, in more detail, how different pathologic conditions may influence brain elastography assessment; (iv) evaluate brain elastography prognostic ability compared to traditional neuroimaging techniques; and (v) confirm brain elastography safety in neonates. It is clear that a more extensive comprehension of neonatal brain USE advantages and limits may open up multiple clinical scenarios in the very next future.

## Figures and Tables

**Table 1:** Studies investigating neonatal BE feasibility in human newborns.

Author (Year)	Study Design	Neonates (n)	Elastographic Technique (Vendor)	Transducer	Feasibility (Yes/No)
Su [10]	Prospective	41	p-SWE (Siemens)	Convex, 1–4 MHz	Yes
Kim [11]	Prospective	21	Strain (Samsung)	Linear, 3–16 MHz	Yes
Albayrak [12]	Prospective	83	2D-SWE (GE)	Convex, 1–6 MHz	Yes
Dirrichs [27]	Prospective	184	2D-SWE (SuperSonic)	Linear, 2–10 MHz	Yes
Garces-Inigo [26]	Prospective	55	2D-SWE (Siemens)	Linear, 4–9 MHz	Yes
El-Ali [29]	Prospective	78	2D-SWE (GE)	Convex, 1–6 MHz	Yes

Note: p-SWE, point shear wave elastography; 2D-SWE, 2-dimensional shear wave elastography.
